# ECD-CDGI: An efficient energy-constrained diffusion model for cancer driver gene identification

**DOI:** 10.1371/journal.pcbi.1012400

**Published:** 2024-08-30

**Authors:** Tao Wang, Linlin Zhuo, Yifan Chen, Xiangzheng Fu, Xiangxiang Zeng, Quan Zou

**Affiliations:** 1 School of Data Science and Artificial Intelligence, Wenzhou University of Technology, Wenzhou, China; 2 College of Computer Science and Electronic Engineering, Hunan University, Changsha, China; 3 Institute of Fundamental and Frontier Sciences, University of Electronic Science and Technology of China, Chengdu, China; Chinese Academy of Sciences Shenzhen Institutes of Advanced Technology, CHINA

## Abstract

The identification of cancer driver genes (CDGs) poses challenges due to the intricate interdependencies among genes and the influence of measurement errors and noise. We propose a novel energy-constrained diffusion (ECD)-based model for identifying CDGs, termed ECD-CDGI. This model is the first to design an ECD-Attention encoder by combining the ECD technique with an attention mechanism. ECD-Attention encoder excels at generating robust gene representations that reveal the complex interdependencies among genes while reducing the impact of data noise. We concatenate topological embedding extracted from gene-gene networks through graph transformers to these gene representations. We conduct extensive experiments across three testing scenarios. Extensive experiments show that the ECD-CDGI model possesses the ability to not only be proficient in identifying known CDGs but also efficiently uncover unknown potential CDGs. Furthermore, compared to the GNN-based approach, the ECD-CDGI model exhibits fewer constraints by existing gene-gene networks, thereby enhancing its capability to identify CDGs. Additionally, ECD-CDGI is open-source and freely available. We have also launched the model as a complimentary online tool specifically crafted to expedite research efforts focused on CDGs identification.

## Author summary

Cancer has become a major disease threatening human life and health. Cancer usually originates from abnormal gene activities, such as mutations and copy number variations. Mutations in cancer driver genes are crucial for the selective growth of tumor cells. Identifying cancer driver genes is crucial in cancer-related research and treatment strategies, as it helps understand cancer occurrence and development. However, the complex gene-gene interactions, measurement errors, and the prevalence of unlabeled data significantly complicate the identification of these driver genes. We developed a new method that integrates an energy-constrained diffusion mechanism with an attention mechanism to uncover implicit gene dependencies in biomolecular networks and generate robust gene representations. Extensive experiments demonstrated that our model accurately identifies known cancer driver genes and effectively discovers potential ones. Furthermore, we analyzed and predicted patient-specific mutated genes, enhancing our understanding of their pathogenesis and advancing precision medicine. In summary, our method offers a promising tool for advancing the identification of cancer driver genes.

## Introduction

Cancer is typically driven by the accumulation of genetic variations, including single nucleotide variations, small insertions or deletions, and copy number variations [1,2]. Gene mutations can lead to activation or inactivation, promoting cancer occurrence and metastasis. Cancer driver genes(CDGs) mutations enable tumor cells to gain selective growth advantages in evading immune cell clearance and drug treatment [3,4]. Therefore, developing methods to identify CDGs is of great significance for cancer pathologic research, as well as the development of cancer diagnosis, treatment, and targeted drugs [5]. The recent advancements in next-generation sequencing technology have helped researchers facilitate the generation of a vast amount of cancer genomic data and classify somatic mutations in common and rare cancer types [6]. Systematically identifying CDGs from large-scale human cancer genomic data remains a significant challenge [7,8].

Many computational methods and tools have been developed to address this challenging issue in the past few years. Traditional computational methods for identifying CDGs can be divided into two main categories: mutation frequency-based and network-based. The mutation frequency-based methods generally assume that mutations in driver genes have a higher probability of being recurrent across samples compared to non-driver genes, thus identifying significantly mutated genes as CDGs [9,10]. The network-based methods consider cancer to result from mutations in multiple genes that collectively play essential roles in cancer-related biological pathways [11,12]. Despite the remarkable achievements of these methods in studying gene variations, there are still some limitations. For example, mutation frequency-based methods often fail to detect driver genes with low mutation frequencies due to the lack of reliable background mutation frequencies. Additionally, when biological networks lack numerous associative relationships or are inundated with a large amount of noise data, this type of method can lead to poor accuracy in identifying driver genes.

Recently, machine learning(ML) techniques, particularly deep learning methods, have achieved tremendous success in identifying CDGs [13–15]. ML-based approaches framethe prediction of driver genes as a classification task, leveraging available data and knowledge to identify driver genes or driver mutations. Typically, these methods utilize a low-dimensional representation of genes’ multi-omic feature vectors, subsequently employing classifiers to identify CDGs. For instance, Parvandeh et al. utilized cancer gene network data to calculate the differences between nodes using the Minkowski distance [16]. They integrated the nearest neighbor algorithm and evolutionary scoring calculation to potential CDGs. Similarly, Han et al. trained an ensemble of models on various types of gene mutations and then applied Poisson’s distribution coupled with Monte Carlo simulations to discover low-background mutation rate CDGs [17]. In another study, Habibi et al. combined mutation data, protein-protein interaction (PPI), and biological process networks. They calculated the score of gene features, engineered a gene-gene network significantly linked to cancer, and performed cluster analysis to study CDGs [18]. However, these traditional machine learning approaches face limitations due to their neglect of complex interactions inherent in gene-gene networks. GNNs offer a promising solution to this constraint. By employing an iterative message passing and aggregation mechanism, GNNs are capable of learning low-dimensional embeddings that capture the complex relationships among genes, based on their interactions within the network [19].

Consequently, GNNs have been instrumental in enhancing the accuracy of CDGs identification [20–22]. For example, the EMOGI model incorporates diverse multi-omics data, including copy number variation, methylation and PPI network to identify CDGs using graph convolutional neural networks (GCNs) [23]. The EMOGI model primarily focuses on a subset of genes in the PPI network, conducting training and evaluation solely at the node level. Building upon this, MTGCN integrates both CDG identification and interaction prediction tasks into a collaborative training framework, thereby improving the precision of CDG prediction [24]. These approaches utilize Chebyshev polynomials within the convolutional layers and separate the embeddings from their neighboring nodes during the aggregation process, which can effectively address the issue of "over-smoothing" often encountered with multiple iterative convolution operations. As a result, these models demonstrate superior performance compared to traditional GCNs [25] and Graph Attention Networks (GATs) [26]. However, these models do have their limitations. Specifically, biomolecular networks are typically highly heterogeneous, a condition primarily attributed to the diversity of genomic data, including gene expression, protein interactions, and metabolite profiles. To our knowledge, the message propagation in most GNN models is often influenced by nodes with high degrees. Consequently, this can lead to the masking or domination of gene features by heterogeneous, highly connected neighbors, which impedes the accurate representation of gene features. To overcome this limitation, Zhang et al. introduced the HGDC model based on graph diffusion models [27]. Initially, HGDC creates an auxiliary graph employing graph diffusion and random walk techniques and jointly trains it alongside the original graph to enhance node representation. Subsequently, it refines the propagation and aggregation mechanisms inherent in GCNs, making the model more suitable for heterogeneous biomolecular networks. Finally, it deploys a multi-layer attention classifier to accurately identify CDGs.

While existing models demonstrate strong performance in identifying CDGs, they have limitations. Most notably, these models often focus solely on the immediate neighborhood of nodes, overlooking potentially complex interdependencies between any two genes. Additionally, data noise introduced by errors in the collection process can further compromise performance. To address these challenges, we propose the ECD-CDGI model, which joins the diffusion process with an attention mechanism to unveil hidden relationships between any two genes and enhance CDG Identification. In summary, the main contributions of this paper are described as follows:

1. ECD-CDGI considers gene interactions as a diffusion process to maintain gene expression globally consistent in terms of the underlying structure while mitigating the effects of noisy data, and for the first time, realizes the combination of energy-constrained diffusion and attention mechanisms to identify CDGs.
2. We design an ECD-Attention encoder based on diffusion processes and attention mechanisms to capture implicit dependencies between genes in biomolecular networks. This approach generates robust gene representations, which are further enhanced by integrating topological information.
3. We introduce a hierarchical attention module to aggregate the output results across each layer during the information propagation process. By augmenting the diversity of node representations, this strategy subsequently improves the predictive accuracy of the ECD-CDGI model.
4. Extensive experiments indicate that the ECD-CDGI model possesses the ability to not only identify known CDGs but also efficiently uncover potential cancer genes. Moreover, compared to the GNN-based approach, the ECD-CDGI model exhibits lower constraints from gene-gene networks, which enhances its ability to identify potential cancer genes.

## Materials and methods

### Materials

The task of identifying CDGs generally draws upon multi-omics data sources including genomics, transcriptomics, proteomics, and metabolomics. The primary workflow entails applying dimensionality reduction techniques to these multi-omics datasets, effectively extracting the low-dimensional representations of genes in the biomolecular network in a reduced dimensional space. Subsequently, the representations of these genes are compared to the representations of known CDGs, enabling the prediction of CDGs. For the scope of this experiment, we utilize a gene set within a 58-dimensional feature space, as cited in the referenced work [27].

The efficacy of the proposed ECD-CDGI model in predicting CDGs was evaluates across three distinct biomolecular network datasets: PathNet [28], GGNet [29], and PPNet [30]. Specifically, the PathNet dataset comprises a network of interlinked biochemical pathways within cells or organisms, incorporating data from both KEGG and Reactome pathways. GGNet is constructed from RNA interaction data, forming a gene-gene network. Meanwhile, PPNet is extracted from the STRING database. Each of these datasets offers a unique perspective, contributing to a comprehensive evaluation of the model’s performance.

In this study, the term "cancer driver genes" refers to genes that are clearly identified and widely recognized for their crucial roles in the initiation and progression of tumors. These genes are categorized as positive samples. Specifically, 711 well-established driver genes were sourced from the NCG database [31], and an additional 85 high-confidence driver genes were identified using the DigSEE tool [32], totaling 796 genes. The positive samples across PPNet, GGNet, and PathNet networks, are derived from these genes. Additionally, drawing on prior findings [23], negative samples were selected based on the following criteria: Exclude genes 1) listed in the NCG database [31], 2) linked to "cancer pathways" from the KEGG database [33], 3) listed in the OMIM disease database [34], 4) predicted by MutSigdb [9] to be cancer-related, 5) with expression patterns similar to known cancer genes. Generally, negative samples comprise genes that are unlikely to be related to cancer. The data used in this study is presented in **Table 1**.

**Table 1:** Statistical details of the datasets.

Network	Number of nodes	Number of edges	Positive	Negative
GGNet	11,309	621,988	708	1101
PathNet	7,699	92,710	598	701
PPNet	11,473	285,843	716	1026

### Problem formulation

The proposed ECD-CDGI model leverages an encoder grounded in both energy-constrained diffusion processes and attention mechanisms. To facilitate a comprehensive understanding of this model and its architecture, we will delineate the foundational principles and associated technologies underpinning the model in the section.

### Energy-constrained diffusion process

The diffusion process can be characterized as the autonomous transition of a system from a high-energy state to a low-energy equilibrium, encompassing intricate node interaction mechanisms. In this context, the diffusivity serves as an indicator of the rate at which signals or energy are transferred. Leveraging the thermodynamic diffusion model [35] and the explicit Euler method [36], we can formulate the state update equation for individual nodes as follows:

$$z_{i}^{t+1}=(1-\tau \sum_{j=1}^{N}S_{ij}^{t})(z_{i}^{t})+\tau \sum_{j=1}^{N}S_{ij}^{t}(z_{j}^{t})$$ (1)

where $S_{ij}^{t}$ denotes the signal propagation rate from node ***i*** to node ***j*** at state ***t***, termed diffusivity, ***Z*** denotes the status of the nodes, and *τ* serves as a balancing parameter. The equation’s first term denotes the embedding of node ***i*** at ***t***-th state, while the second term aggregates the embeddings of all other nodes. Importantly, the updated status of node ***i*** is effected by all other nodes, and the influence of each node-node pair is dynamically updated as well [37]. At this moment, Eq 1 bears a close resemblance to the attention mechanism employed in transformers, with the diffusivity matrix *S*^*t*^ corresponds to the node-node attention matrix at the ***t***-th layer.

To ensure that node representations absorb both global and local information, energy constraints are integrated into Eq 1 with the intent that node representations in each state contribute to a reduction in overall energy, steering the system toward a more stable state. Consequently, Eq 1 can be reformulated as follows:

$$z_{i}^{t+1}=\left(1-\tau \sum_{j=1}^{N}S_{ij}^{t}\right)(z_{i}^{t})+\tau \sum_{j=1}^{N}S_{ij}^{t}(z_{j}^{t}),$$

$$s.t.\;E(Z,t,\kappa )\leq E(Z,t,\kappa )\;Z^{0}=X,$$ (2)

where

$$E(Z,t,\kappa )={\left\|Z-Z^{t}\right\|}_{F}^{2}+\omega \sum_{i,j}\kappa ({\left\|z_{i}^{t}-z_{j}^{t}\right\|}_{F}^{2}).$$ (3)

where the first term encapsulates the local consistency of each node relative to its current state, while the second term embodies the global consistency across all nodes, and *ω* denotes the balance parameter. Here, *κ* stands for a monotonically increasing concave function, strategically designed to lessen the penalty imposed on node-node pairs that exhibit significant differences. This expects to enhance the diversity of node representations within the network.

Directly solving for the diffusivity matrix *S*^*t*^ poses a considerable challenge. Fortunately, an analysis of energy constraint function reveals that the energy decline and diffusion process maintain a certain degree of consistency. Consequently, a diffusion process guided by energy constraints can be established as follows:

$${\hat{S}}_{ij}^{t}=\frac{\omega_{ij}^{t}}{\sum_{k=1}^{N}\omega_{ij}^{t}},\omega_{ij}^{t}=\frac{\partial \kappa (z^{2})}{\partial z^{2}},\;z^{2}={\left\|z_{i}^{t}-z_{j}^{t}\right\|}_{2}^{2}.$$ (4)

where $\omega_{ij}^{t}$ symbolizes the similarity between nodes ***i*** and ***j***. By incorporating nonlinear functions like the ∂ function to compute node-node similarities, thereby enhancing the model’s expressive capabilities. Then the computation for $\omega_{ij}^{t}$ can be empirically defined as follows:

$$\omega_{ij}^{t}=f({\left\|{\hat{z}}_{i}^{t}-{\hat{z}}_{j}^{t}\right\|}_{2}^{2})=\frac{1}{1+\exp(-(z_{i}^{t}{)}^{Τ}(z_{i}^{t}))}.$$ (5)

In this way, the diffusivity serves as a measure of the influence between any two nodes and can also be interpreted as attention of each node-node pair. This insight informs the architecture of encoders built on energy-constrained diffusion processes and attention mechanisms.

### Model architecture

**Fig 1:**
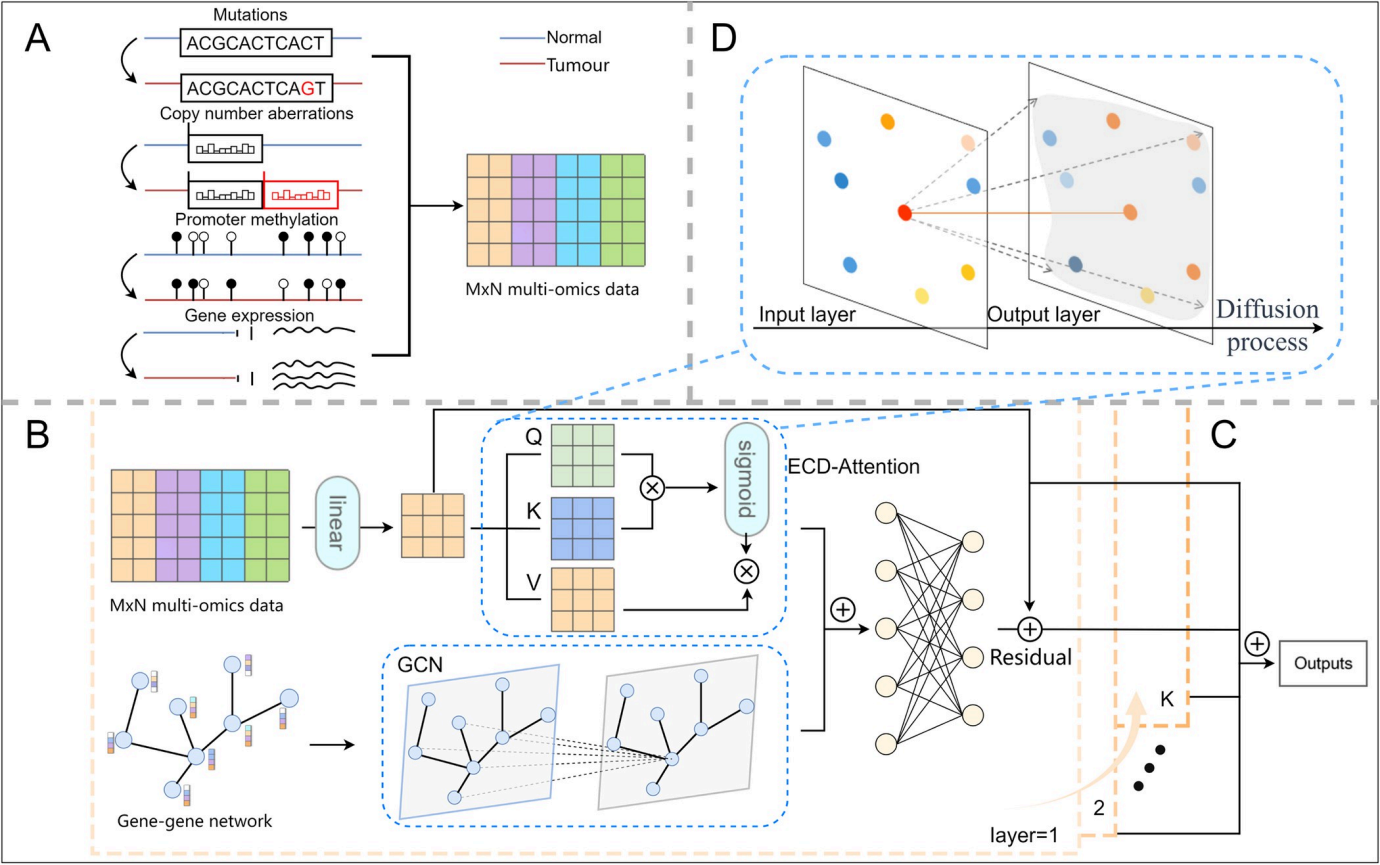
The architecture of the ECD-CDGI model mainly includes three principal modules: **(A)** Data Module, **(B)** Encoder Module, and **(C)** Multi-layer Attention Module. **(A)** The Data Module primarily contains the initial feature vectors and topological architecture of gene nodes within the biomolecular network. **(B)** The Encoder Module is consisting of three key components: a newly-conceived ECD-Attention encoder based on energy-constrained diffusion process **(D)**, a GNN encoder, and a residual connection. **(C)** Employing a hierarchical structure, the Multi-layer Attention Module integrates data across various layers to formulate a comprehensive node representation, which is then used to identify CDGs effectively. **(D)** The energy-constrained diffusion process.

**Fig 1** illustrates the architecture of the ECD-CDGI model, comprising primarily three modules: the Data Module, the Encoder Module (including ECD-Attention encoder, GNN encoder and Residual connection), and the Multi-layer Attention Module. To enrich the datasets, both the initial feature vectors of gene nodes in the biomolecular network and the network’s topological structure were extracted, as detailed in the materials section. To address the challenges posed by noisy observational data and latent dependencies among nodes within biomolecular networks, we design a novel encoder, termed ECD-Attention. This encoder is ground in energy-constrained diffusion processes and attention mechanisms. **Fig 1(D)** illustrates the energy-constrained diffusion process, wherein the energy (information) from each node is distributed to all other nodes in the network, ensuring that the state of each node is influenced by that of every other node. Simultaneously, a GNN encoder is used to mine the topological structure of the biomolecular network, thereby augmenting gene representations. Employing a multi-layer attention mechanism, the proposed model assimilates information across multiple scales to efficiently identify CDGs.

The ECD-CDGI model employs a automatic approach to identify CDGs, including several key stages: Initially, the multi-omics data information of genes within the biomolecular network is fed into the ECD-Attention encoder, while concurrently, the topological information is input into the GNN encoder. The features extracted from both encoders are then concatenated, followed by residual connections and layer normalization operations. Subsequently, leveraging the message propagation mechanism, the encoding process undergoes multiple iterations, generating multiple sets of gene representations. Ultimately, the multi-layered data is fused utilizing the hierarchical attention module, resulting in the final node representations. These comprehensive representations are then employed to predict CDGs.

#### ECD-Attention encoder

Building on the insights gained from the Preliminary Section, the diffusion process is governed by energy constraints, which aim to reduce the overall system energy during diffusion, thereby stabilizing the system. And inspired by previous work [38], we introduce an ECD-Attention encoder that incorporates both energy-constrained diffusion and attention mechanisms. This encoder is crafted to ensure the local consistency of each gene node’s current state during the information propagation process that is similar to the diffusion process, while also preserving global consistency with other gene nodes in the biomolecular network. Notably, the encoder effectively dampens the impact of data noise and reveal latent interdependencies between genes. The following is a detailed presentation of the relevant principles and steps.

First, the multi-omic features *X*∈*R*^*N*×58^ of genes are mapped into a low-dimensional embedding space through a fully connected layer.

$$Z=\sigma (W_{I}X+b_{I})$$ (6)

where *W*_*I*_ and *b*_*I*_ serve as trainable parameters, while *σ* denotes the nonlinear activation function, specifically ***ReLU*** in this context. And the resulting ***Z*** serves as the initial embedding *Z*^0^.

Leveraging attention-based information propagation principles, the gene embedding matrix ***Z*** undergoes multiple iterations:

$$K^{t}=W_{K}^{t}Z^{t},Q^{t}=W_{Q}^{t}Z^{t},V^{t}=W_{V}^{t}Z^{t},$$ (7)

where *K*^*t*^, *Q*^*t*^ and *V*^*t*^ serve as the transformed ***Key***, ***Query***, and ***Value*** matrices at ***t***-th layer, respectively, while $W_{K}^{t},\;W_{Q}^{t}$ and $W_{V}^{t}$ are their corresponding transformation weight matrices.

Leveraging the energy-constrained diffusion and attention mechanisms, the diffusivity matrix in the diffusion process can be reinterpreted as an attention matrix for gene-gene pairs. Echoing the principles outlined in the Preliminary Section, a straightforward dot-product method is employed to quantify the similarity between any two genes. Furthermore, within the energy-constrained diffusion process, the node state update rule considers the state of all nodes, meaning each node’s state is influenced by every other node. Node state updates are executed by integrating the complete node-node similarity matrix with the value vector. Clearly, this approach is well-suited for the Transformer architecture. In the Transformer architecture, node-node attentions resemble the signal propagation rate *S* observed in energy-constrained diffusion processes. This process normalizes the similarity between nodes using dot product and sigmoid operations.

To serve as the attention function, the sigmoid activation is utilized to constrain the input vector within the range of [0,1], ensuring that the resulting attention weights are non-negative:

$$R^{t}=\sigma (Q^{t}{\left(K^{t}\right)}^{Τ}),$$ (8)

where *R*^*t*^ denotes the similarity matrix. Define *diag*^−1^ denotes the inverse of the diagonal matrix, then the gene-gene attention $R_{attention}^{t}$ and gene embedding *P*^*t*^ of the ***t***-th layer can be calculated:

$$R_{attention}^{t}=diag^{-1}(A^{t}1),P^{t}=R_{attention}^{t}A^{t}V^{t},$$ (9)

### GNN encoder

Utilizing the ECD-Attention encoder, embeddings that based on gene-gene attention are derived from the multi-omics data. At this stage, the model hasn’t yet integrated the topological information of the biomolecular network. However, such topological structures serve as crucial auxiliary information, shedding light on the local distribution of gene nodes within the network. Consequently, incorporating this topological data is of importance. Conveniently, this study employs a GCN as the encoder, and the GCN encoder updates the embeddings according to the following equation:

$$H^{t}=D^{-1/2}AD^{-1/2}V^{t},$$ (10)

where ***A*** denotes the adjacency matrix of the gene-gene graph, and ***D*** represents its diagonal matrix. *H*^*t*^ denotes the embedding matrix of the ***t***-th layer of the GNN encoder.

### Residual connection

To integrate both the latent gene-gene interdependencies and the topological features within gene networks, the output embeddings from the ECD-Attention and GCN encoders are concatenated:

$$Z^{t+1}=W_{d}^{t}(P^{t}\|H^{t}),$$ (11)

where **||** denotes the concatenation operation, and *W*_*d*_ serves as the parameter matrix for a learnable linear transformation. To facilitate more effective training, we introduce residual connections:

$$Z^{t+1}=LayerNorm(\beta Z^{t+1}+(1-\beta )Z^{t}),$$ (12)

where *β* represents the balance parameter, and ***LayerNorm*** represents the layer normalization.

### Multi-layer attention

During the course of information propagation, genes within the biomolecular network are subject to evolving states at each computational stage. Capturing the evolving representations of these genes at different states is essential. Traditional approaches, such as conventional GNN models, often rely solely on the output from the final layer. In biomolecular networks, the iterative propagation of information often leads to increasingly uniform gene representations, thereby limiting the model’s representation capacity and its ability to detect nuanced local variations. To address these challenges, our proposed ECD-CDGI model incorporates a hierarchical attention mechanism [39] to aggregate multi-layer node embeddings of genes, effectively mitigating the aforementioned issues. During information propagation, the model assigns learnable attention weights (denoted as *α*^0^,*α*^1^,…,*α*^*T*^) to each layer’s gene representation, signifying the layer’s relative importance.

$$M=\sum_{t=0}^{T}\alpha^{t}Z^{t}W_{Z}^{t},$$ (13)

where ***M*** denotes the final representations of genes, $W_{Z}^{t}$ serves as the tunable weight matrix associated with *Z*^*t*^, while ***T*** designates the total number of layers in the model. The classification loss for the model is then computed using the binary cross-entropy loss function as follows:

$$L=-\frac{1}{N}[y_{v}\;\log\left(M_{v}\right)+(1-y_{v})\log\left(1-M_{v}\right)],$$ (14)

where *y*_*v*_ denotes the actual label corresponding to gene ***v***, *M*_*v*_ signifies the model’s predicted score for gene ***v***, and ***N*** represents the total count of genes included in the training dataset.

## Results

To evaluate the efficacy of the ECD-CDGI model, we execute multiple sets of experiments using publicly available datasets. Initially, we engage in comparative analyses against state-of-the-art methods for CDG identification to validate the model’s superior capabilities. Subsequently, we design a series of ablation experiments to evaluate the individual contributions of various modules within the ECD-CDGI architecture. In the final phase, we delve into specific case studies and explore the scalability prospects of our proposed model.

### Implementation detail

This study was conducted using the Python and Pytorch frameworks, focusing on parameters associated with the ECD-Attention encoder, GCN encoder, and multi-layer attention module, along with various hyperparameters. Genomic data served as the initial input for the model, with its dimensionality set at 58. In the ECD-Attention encoder, the transformation weight matrices are preset to a dimension of 100. The multi-layer attention module is configured with four layers by default, with each layer’s initial weight preset at 0.5. Both the ECD-Attention and GCN encoders are integrated across 4 layers. Other hyperparameters include a hidden layer dimension of 100, 100 training rounds, a default learning rate of 0.001, and Adam as the optimizer.

### Comparison experiment

We designed a series of benchmarking experiments across three publicly accessible datasets GGNet, PathNet, and PPNet, to compare the performance of our ECD-CDGI model with six other methods. These comprise three advanced CDG prediction models EMOGI [23], MTGCN [24], and HGDC [27], as well as three conventional GNN models GCN [25], GAT [26], and ChebNet [40]. To ensure a level playing field, each method was fed the same feature matrix corresponding to biomolecular networks. We carried out ten times of 5-fold cross-validation for each model. The final performance metrics, represented by the average ***AUC*** and ***AUPR*** scores, are presented in **Table 2**.

**Table 2:** Performance comparison of multiple models on three datasets (%).

Comparative Algorithms	GGNet		PathNet		PPNet	
	AUC	AUPR	AUC	AUPR	AUC	AUPR
GCN [25] GAT [26] EMOGI [23] MTGCN [24] HGDC [27] ChebNet [40]	49.28 50.93 79.90 81.77 82.60 82.85	43.17 42.11 74.10 75.29 77.35 77.67	72.58 73.30 80.73 84.03 84.10 85.00	71.20 66.04 78.07 82.40 82.90 83.27	76.29 73.35 80.02 82.08 83.35 84.08	70.56 62.44 74.43 75.24 79.31 80.08
ECD-CDGI	**84.15**	**79.19**	**86.24**	**85.29**	**86.21**	**82.84**

As reflected in **Table 2**, EMOGI, MTGCN, HGDC, ChebNet, and our proposed ECD-CDGI model all demonstrated commendable performance in the task of identifying CDGs. The GCN and GAT models lagged behind in terms of effectiveness. Notably, the EMOGI, MTGCN, HGDC, and ChebNet algorithms all employ Chebyshev polynomials to perform convolution operations. During the message propagation and aggregation phases, these models differentiate between neighboring nodes and the nodes themselves, thereby mitigating the performance degradation typically induced by over-smoothing. Building upon this, the HGDC model incorporates an auxiliary network crafted using graph diffusion technology and aims to enhance predictive accuracy through joint training with the original network. However, it’s noteworthy that HGDC’s performance remains on par with, or even slightly underperforms, the original ChebNet model. This suggests that the auxiliary network generated through graph diffusion techniques may introduce an element of unpredictable noise.

**Fig 2:**
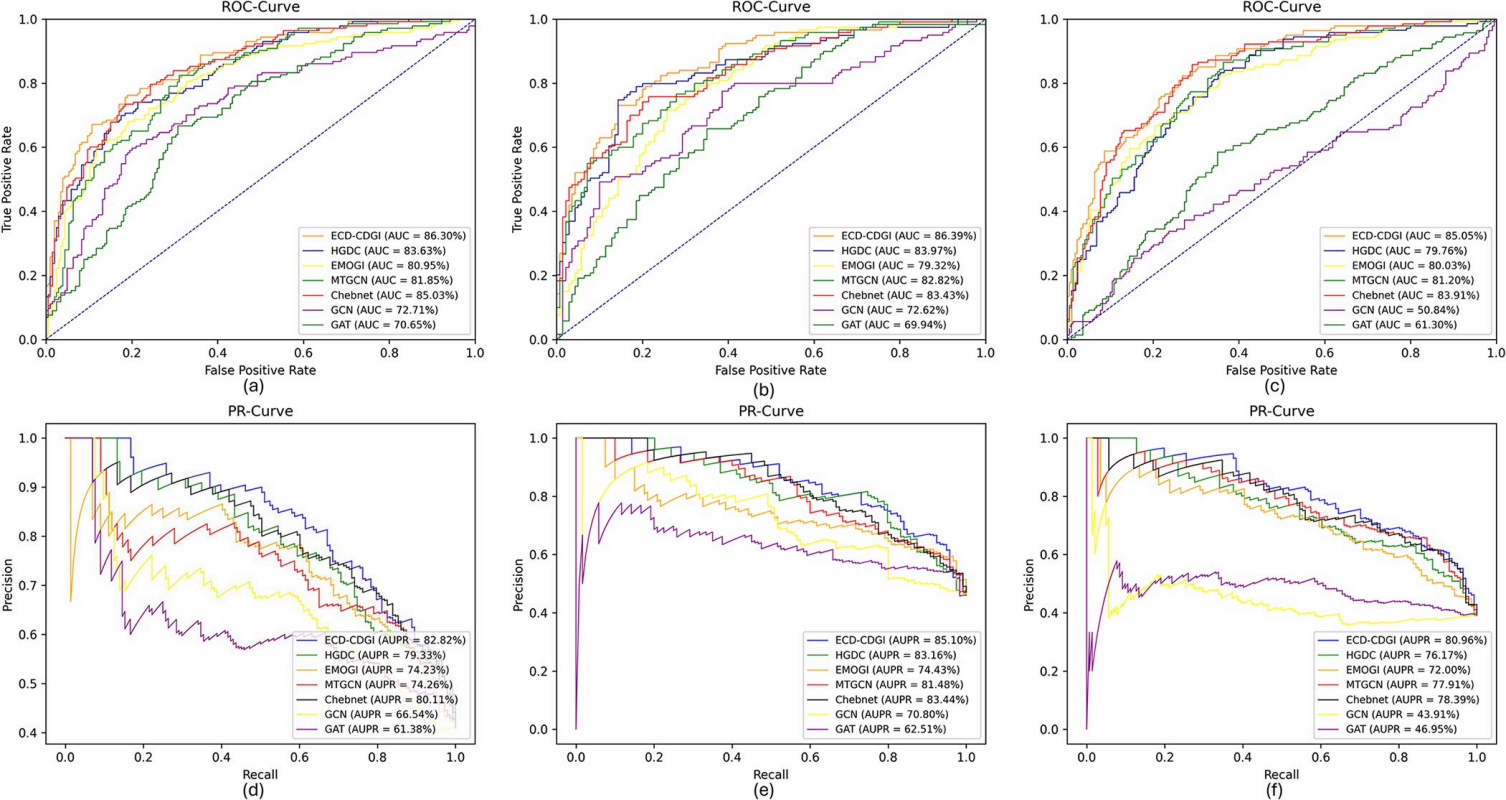
ROC curves for multiple models on (a) PPNet, (b) PathNet, and (c) GGNet datasets; PR curves for (d) PPNet, (e) PathNet, and (f) GGNet datasets.

It’s important to highlight that our proposed ECD-CDGI model outperformed all competitors across all datasets. It led the second-best performing model by margins of 1.30%, 1.24%, and 2.13% in the ***AUC*** index, and by 1.57%, 2.02%, and 2.76% in the ***AUPR*** index. These results underscore the efficacy of the ECD-Attention encoder, which is grounded in energy-constrained diffusion and attention mechanisms. This encoder is adept at unveiling the complex interdependencies among genes. When combined with the GCN encoder to harness the topological information of the gene-gene network, it substantially enhances the quality of node representation. As illustrated in **Fig 2**, we plotted the ***ROC*** and ***PR*** curves for each model on three datasets. The curves for ECD-CDGI model consistently outpace other models and demonstrate remarkable stability. This provides additional validation that the ECD-CDGI model is both efficient and reliable in identifying CDGs.

### Ablation experiment

This section aimed to evaluate the individual contributions of four key modules within the ECD-CDGI model: the ECD-Attention encoder, the GCN encoder, the residual connection, and the multi-layer attention mechanism. To facilitate this, we conduct ablation experiments across three datasets GGNet, PathNet, and PPNet, while holding other variables constant. The term ’w/o ECD-Att’ denotes a model configuration that removes the ECD-Attention encoder, relying solely on the GCN encoder. Conversely, ’w/o GCN’ signifies a setup where the GCN encoder is excluded, with only the ECD-Attention encoder in place. And ’w/o Residual’ means that the residual connection module has been removed, while ’w/o multi-Att’ implies that the model delete the multi-layer attention mechanism and employs only the encoder’s final layer output for both training and prediction.

We performed ten times of 5-fold cross-validation experiments for each model configuration across three datasets. The results are summarized as average values for the ***AUC*** and ***AUPR*** metrics, as detailed in **Table 3**. Generally speaking, any version of the ECD-CDGI model that omits one of its key components, whether it’s the ECD-Attention encoder, GCN encoder, residual connection, or multi-layer attention mechanism, experiences a decline in performance. The ECD-Attention encoder captures global information, revealing potential dependencies between indirectly connected genes. The GCN encoder receives information from neighboring nodes and effectively propagates messages based on gene interactions. Residual connections maximize the retention of original features during iterations, preventing the loss of information from nodes in previous layers. The multi-layer attention mechanism automatically learns weights and integrates node representations across weighted iterations, enhancing model performance.

**Table 3:** Results of ablation experiments on three datasets (%).

Comparative Algorithms	GGNet		PathNet		PPNet	
	AUC	AUPR	AUC	AUPR	AUC	AUPR
w/o GCN w/o Residual w/o ECD-Att w/o multi-Att	83.96 82.38 83.25 83.16	78.94 77.50 78.30 78.18	84.75 84.86 86.01 85.87	83.70 83.84 85.24 84.67	83.72 83.10 85.84 85.97	80.20 79.33 82.76 82.60
ECD-CDGI	**84.15**	**79.19**	**86.24**	**85.29**	**86.21**	**82.84**

Diving into details, the model’s performance declines slightly on the GGNet dataset when the GCN encoder is omitted, whereas a more substantial decrease is observed on both the PathNet and PPNet datasets. Intriguingly, this pattern is reversed when the ECD-Attention encoder is omitted. This suggests that the high heterogeneity and complex topological structure of the GGNet dataset may make it difficult for GCNs to effectively capture the intricate relationships and dependencies within the data. The finding also highlights the ECD-Attention encoder’s ability to uncover latent interdependencies among genes, thus boosting the model’s overall performance. Most notably, the model experiences its poorest performance when the Residual module is omitted, indicating its critical role in mitigating the over-smoothing arising during information propagation. It is noteworthy that the Residual module serves as a pivotal element within the ECD-Attention encoder, supplying essential information about the node’s current state during the energy-constrained diffusion process.

### Skewed distribution and enrichment analysis

We conducted extensive experiments and analyses across the GGNet, PPNet, and PathNet datasets to evaluate the capability of our proposed ECD-CDGI model to identify previously unknown CDGs. To mitigate the influence of random variables, we ran the ECD-CDGI model through 100 iterations on each of these datasets, thereafter analyzing the predicted gene scores.

**Fig 3:**
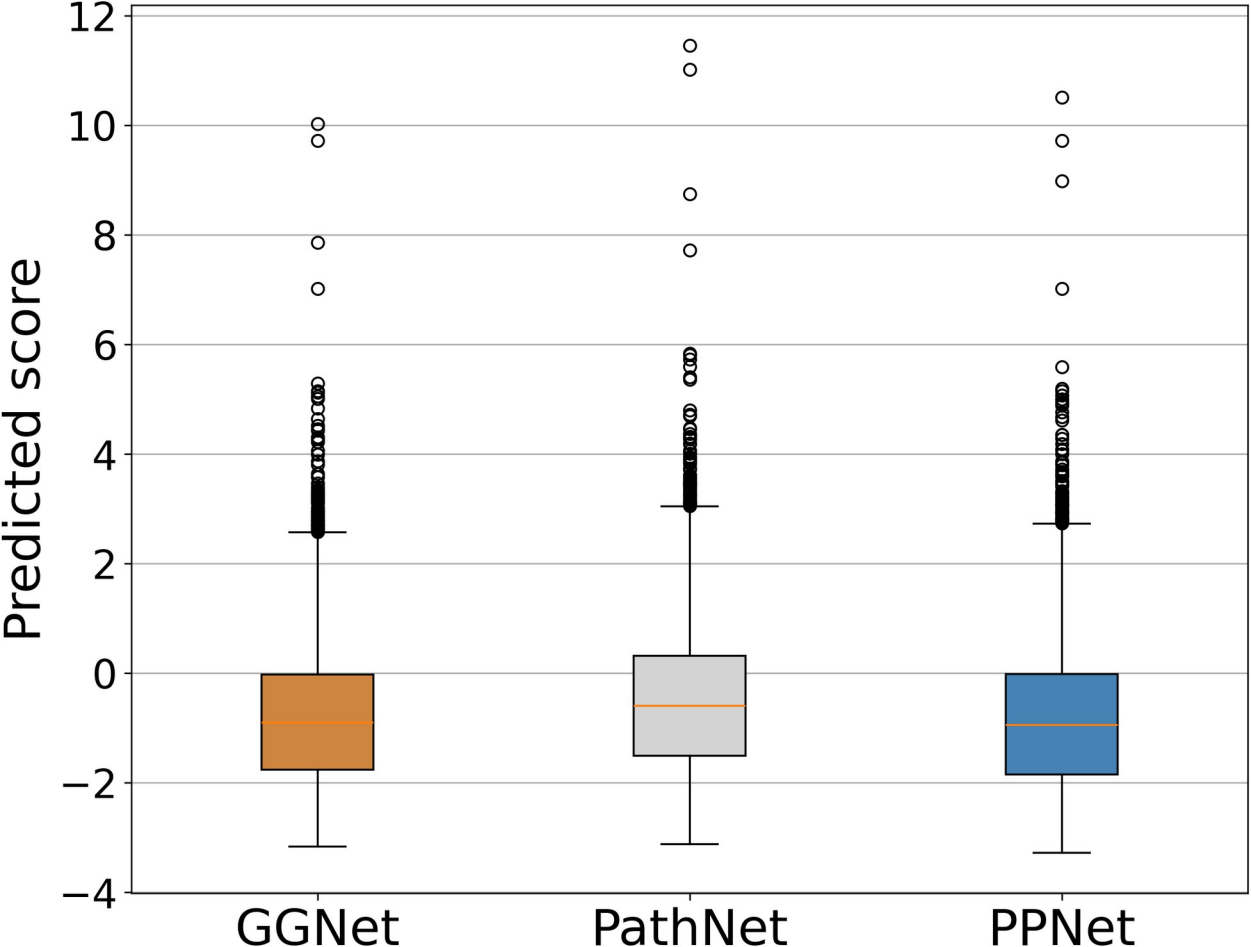
Predicted scores for genes on three datasets using ECD-CDGI model.

As illustrated in **Fig 3**, the gene scores predicted by the ECD-CDGI model across all datasets exhibit a positive skewness. A scant number of genes gain conspicuously high scores, deviating from the central cluster of the data, while the majority of gene scores hover between -2 and 0. This is likely attributable to the fact that the overwhelming majority of genes are not CDGs, resulting in only subtle variations in their scores. In contrast, the outliers in the dataset suggest a small subset of genes with markedly higher scores, pointing to a heightened likelihood of them being CDGs. Overall, the ECD-CDGI model demonstrates a robust ability to differentiate these CDGs from other non-CDGs.

**Fig 4:**
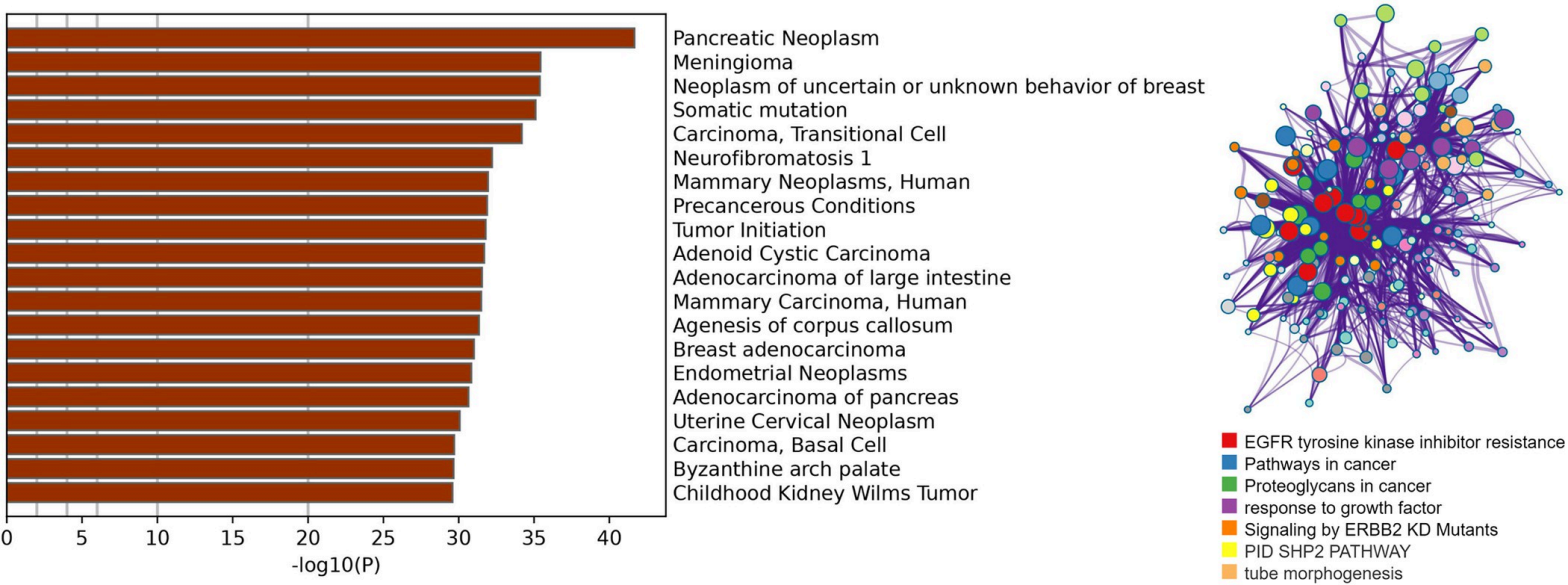
(a) Results of gene enrichment analysis for various cancers using the ECD-CDGI model; (b) Enrichment analysis leveraging KEGG pathways and GO biological processes.

We selected and merged the top 100 genes with the highest scores from three networks, resulting in a total of 178 unique genes. This was done to assess the ECD-CDGI model’s ability to recognize these genes. With reference to the DisGeNET database [41], these highly scored genes were further enriched. In **Fig 4(A)** each bar on the left represents a different cancer category; the length of the bar indicates the statistical significance of the gene set linked to that disease. A higher -log10(P) value correlates with a lower p-value, suggesting a stronger association between the gene set and the disease. These results suggest that these high-scoring genes are significantly associated with various diseases, predominantly cancers, particularly pancreatic tumors. To further investigate these genes, we conducted pathway and process enrichment analyses using KEGG pathways, GO biological processes, and other resources, categorizing the genes into clusters based on similarities. In **Fig 4(B)**, on the right, genes are depicted as nodes in different colors, each color representing a distinct enriched pathway. The size of each node correlates with the level of gene enrichment in the corresponding pathway. Purple lines between nodes indicate interactions among genes or the biological processes in which they participate. Of these, 44 genes (24.72%) showed significant enrichment in the "Cancer Pathway" (KEGG Pathway). These genes are likely pivotal in the genesis and progression of tumors. This underscores the capacity of the ECD-CDGI model to identify CDGs accurately, thereby aiding in the elucidation of cancer initiation and progression mechanisms as well as informing relevant treatment strategies.

### Identifying new cancer genes

**Fig 5:**
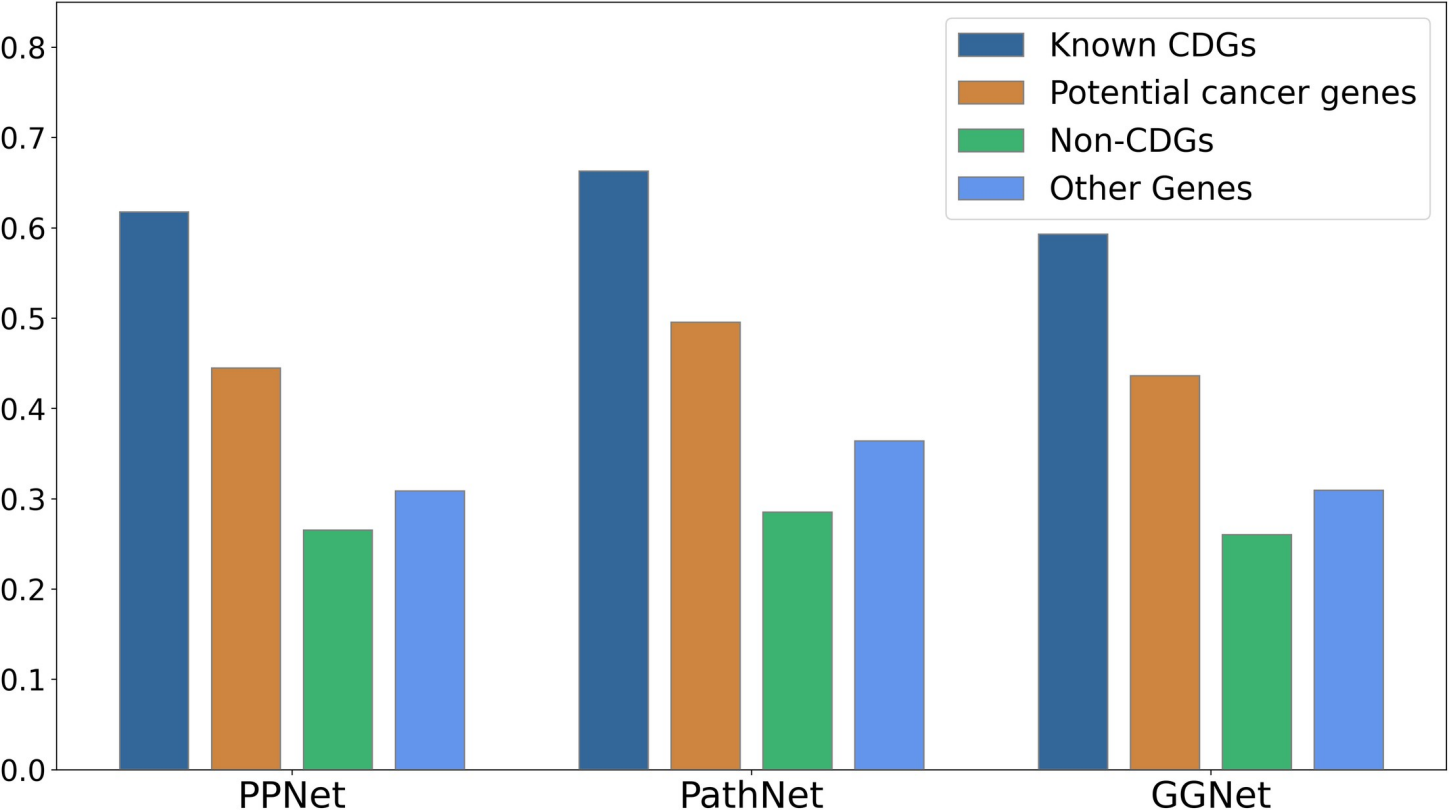
Predicted probabilities for four types of genes on three datasets using ECD-CDGI model.

To validate the efficacy of the ECD-CDGI model in identifying novel cancer genes, we conducted targeted experiments. Specifically, we computed the average prediction probabilities for four categories of genes: known CDGs, non-CDGs, a set of potential cancer genes from the ncg7.1 database, and other genes across the GGNet, PathNet, and PPNet datasets. The results detailed in **Fig 5** reveal that known CDGs garnered the highest average predicted probabilities, while non-CDGs received the lowest. This underscores the ECD-CDGI model’s capability to accurately differentiate between CDGs and non-CDGs. Intriguingly, the average predicted probability for potential cancer genes was also markedly higher than that for non-CDGs and other genes. This suggests that the ECD-CDGI model is not only proficient in identifying known CDGs but is also adept at uncovering potential cancer genes.

### Case analysis

**Fig 6:**
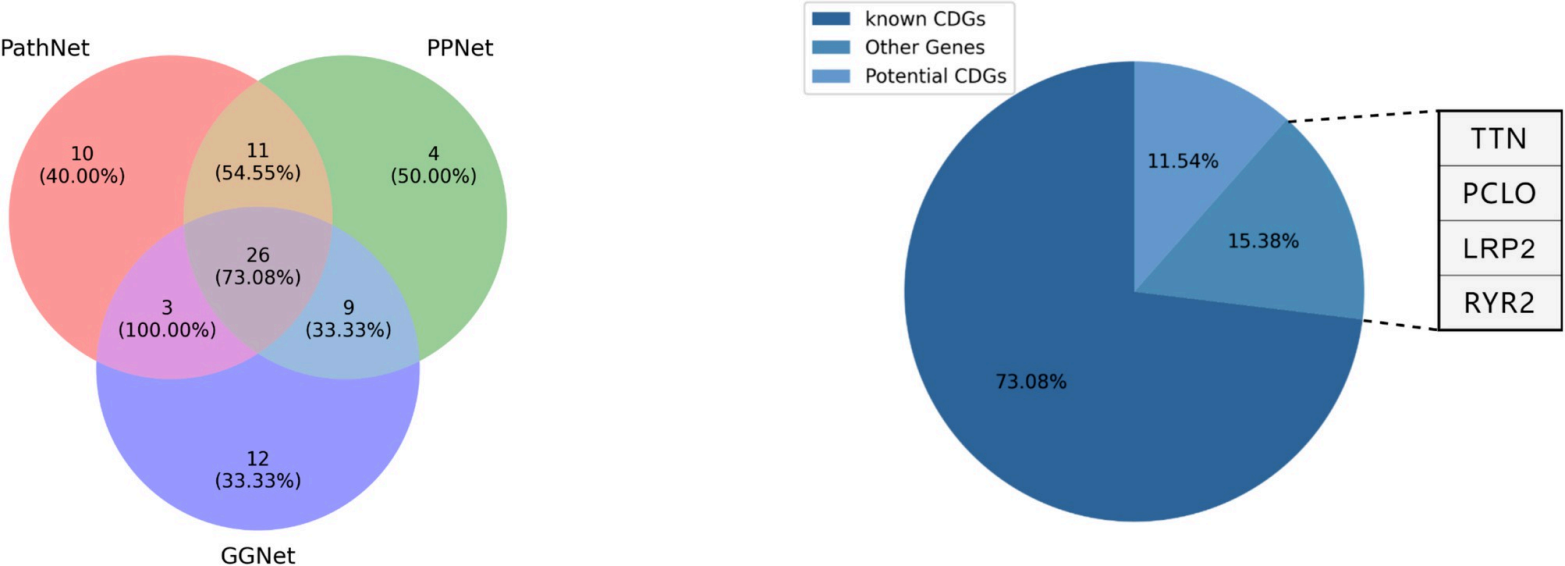
(a)Venn diagram illustrating the quantity and proportion of CDGs identified by ECD-CDGI model across three datasets. (b)Pie chart showing the proportion of known CDGs, cancer-related genes, and other genes identified as CDGs by the ECD-CDGI model on three datasets.

We undertook a comprehensive comparative analysis to evaluate the adaptability of the ECD-CDGI model across diverse datasets. Specifically, we selected the top 50 genes with predictive scores from the GGNet, PPNet, and PathNet datasets, and then quantified the number and percentage of CDGs involved. These findings are visually represented in **Fig 6(A)** through a Venn diagram. Interestingly, the likelihood of identifying a CDG that is unique to a single dataset is notably lower than discovering one that appears across multiple datasets. This observation indicates that genes scoring highly across various datasets are more likely to be CDGs. It’s important to acknowledge that due to inherent constraints in each dataset, such as the presence of noisy data, the complexity of multi-omics data, and variations in gene topological networks, predictive inaccuracies may occur within the ECD-CDGI model. To mitigate these limitations, a cross-dataset analysis can be performed to enhance the precision in identifying CDGs.

Additionally, we delved into the analysis of CDGs that were consistently identified across all three datasets. As depicted in **Fig 6(B)**, out of the 26 genes analyzed, 19 were classified as CDGs, making up 73.08% of the total. Three genes, although not defined as CDGs, were listed as cancer-related in the ncg7.1 database, and constituted 11.54% of the sample. Four other genes TTN, PCLO, LRP2, and RYR2, accounted for the remaining 15.38%. While these genes are not cataloged in the ncg7.1 database, existing literature [42–44] suggests their significant relevance to cancer.

**Fig 7:**
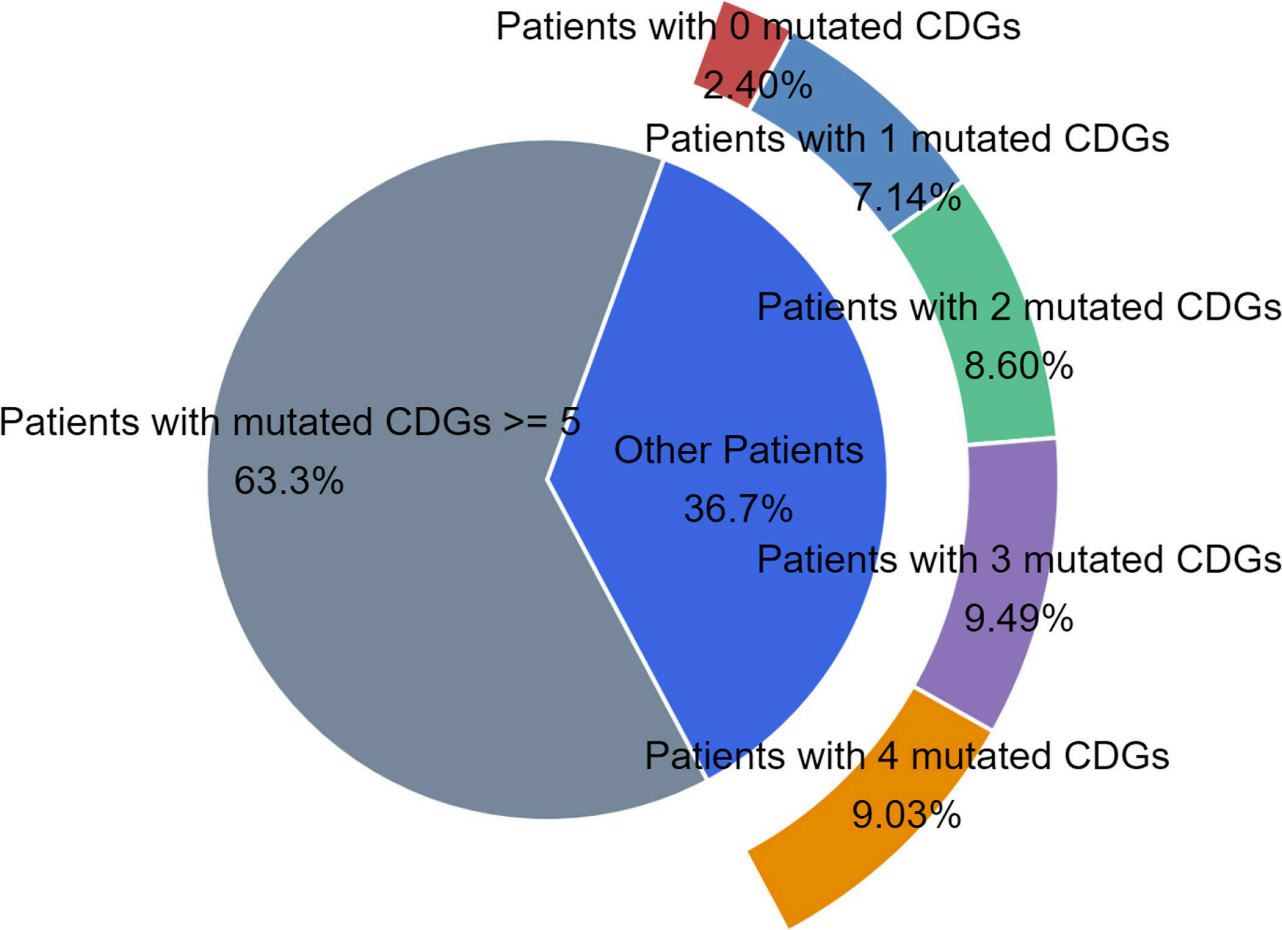
Gene mutation distribution for the specified patients.

To investigate patient-specific CDGs, we gathered and assessed patient-specific data using the ECD-CDGI model. Mutant genes with higher prediction scores are more likely to be specific driver genes, potentially accelerating cancer progression. Specifically, we utilized the Xena tool [45] to collect somatic mutation data from 5776 patients across 14 cancer types in the TCGA database [45]. Initially, we screened and retained genes present in the GGNet, PathNet, and PPNet networks from the patients’ mutant gene data. Building on this, we selected 5535 patients with five or more mutant genes for further analysis. We quantified the mutant genes of each patient (see **Fig 7**) and observed that some patients had fewer than five cancer driver genes, with 2.40% of patients lacking any cancer driver genes in their mutations. Prior studies suggest that having five or more cancer driver genes may correlate with individual cancer development [46]. Therefore, identifying patients’ specific CDGs is crucial for targeted treatment.

**Fig 8:**
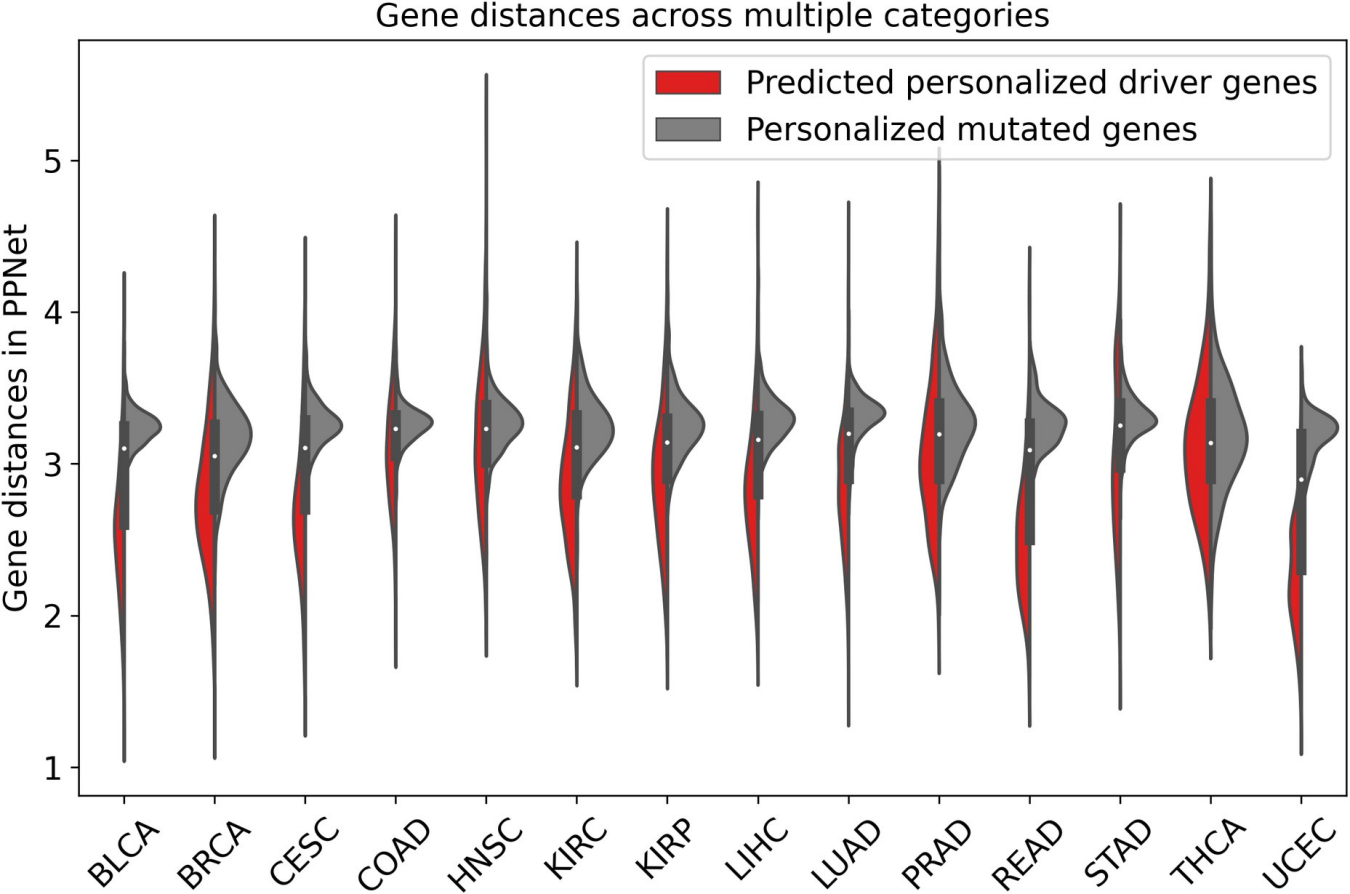
Comparative distances of predicted driver genes vs. mutated genes in various cancers.

In this study, we assessed the ECD-CDGI model’s efficacy in identifying patient-specific CDGs for mutant genes, alongside relevant analyses. Specifically, the model was trained using omics data from 14 cancer types on three biomolecular networks: GGNet, PathNet, and PPNet. For each type of cancer, the model generated three predictive gene ranking lists. For each patient, the Rank algorithm [47] was employed to merge the three gene rankings into a consolidated final list. Subsequently, the top five mutant genes from the final ranking were selected as the specific CDGs for each patient. As illustrated in **Fig 8**, within the PPNet network, the shortest distances between the identified driver genes were notably shorter than those between the mutant genes prior to screening. This suggests that the identified CDGs are closely interconnected, likely cooperating within shared biological pathways or functional modules. This tight linkage intensifies their impact on tumor formation, potentially accelerating tumor progression and malignancy.

In subsequent analyses, we focused on the top 500 genes with the highest prediction scores across the GGNet, PPNet, and PathNet datasets. After removing well-established CDGs, we consider the remaining genes as potential cancer genes. We then probed whether a relationship exists between these potential cancer genes identified by the ECD-CDGI and their connectivity to known CDGs.

**Fig 9:**
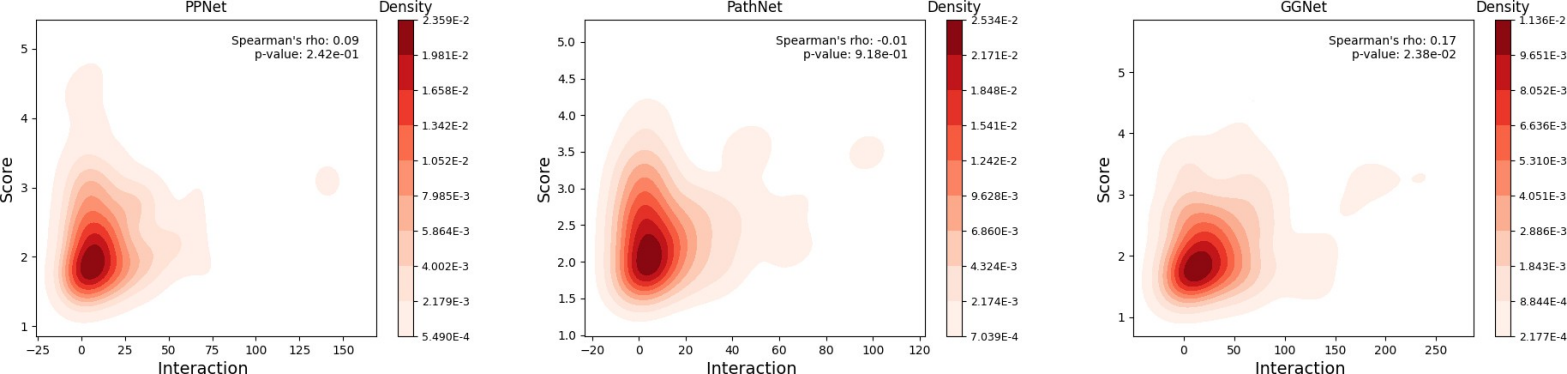
Correlation between a gene’s prediction score and the number of its interactions linked to known CDGs on three datasets.

As illustrated in **Fig 9(A)** and 9**(B)**, for the PPNet and PathNet datasets, the Spearman correlation coefficients are both below 0.1, and the p-values significantly exceed the 5% significance threshold. This indicates only a marginal correlation. **Fig 9(C)** reveals that in the GGNet dataset, the Spearman correlation coefficient is 0.17, with a p-value of 0.0238, falling below the 0.05 threshold, signifying a slight but statistically significant positive correlation between the two variables. These results suggest that the potential cancer genes identified by the ECD-CDGI model exhibit a lower degree of reliance on known CDGs. Importantly, this implies that the ECD-CDGI model is less constrained by existing gene-gene networks in identifying potential cancer genes. As a result, it is better suited for the discovery of novel cancer genes, a task that proves challenging for methods based on GNNs.

## Discussion and Conclusion

This study investigates the pivotal importance of identifying CDGs for both cancer research and clinical treatment, and evaluates various methodologies geared towards this purpose. While existing machine learning and deep learning techniques are indeed effective, they come with inherent limitations. Most notably, these methods often overlook the complex interdependencies between any two genes and may be compromised by noisy data, a byproduct of data collection oversights.

To address these shortcomings, we introduce the ECD-CDGI model, which incorporates a energy-constrained diffusion process and an attention mechanism. By combining with GNNs and multi-layer attention techniques, our model offers a robust tool for identifying CDGs. Our specially designed ECD-Attention encoder not only uncovers the complex global interrelationships between any two genes but also captures nuanced local information to individual gene nodes. Additionally, we integrate residual connections within the model’s layers to mitigate the performance degradation caused by over-smoothing during inter-layer information propagation. Employing GNN technology, the ECD-CDGI model is capable of extracting topological information from gene-gene networks and leverages a multi-layer attention mechanism for predicting CDGs. Comparison and ablation experiments conducted on public datasets confirm the model’s superior performance. We anticipate that the ECD-CDGI model will assume a significant role in cancer research and treatment protocols, offering researchers an efficient tool for understanding the mechanism of cancer development.

Despite its efficacy in CDG prediction, the ECD-CDGI model has certain limitations. Firstly, the presence of missing or erroneous links in biomolecular networks can compromise the model’s performance. Excessive errors or missing links can mislead the learning process and diminish the model’s accuracy. Secondly, while graph neural networks utilize the topological information in biomolecular networks effectively, the absence of comprehensive omics data still impacts their performance. In practical applications, critical omics data, including gene expression, protein interactions, and metabolite profiles, are often incomplete or unavailable. This lack of data can prevent the model from fully understanding gene network interactions, potentially misleading its learning process. Additionally, integrating and synergizing various types of omics data presents challenges due to differing data characteristics and noise levels, where improper handling could impair the model’s performance. To address these issues, future work will focus on mitigating the identified problems. Firstly, we plan to employ debiasing and sampling techniques to minimize the effects of erroneous or incomplete data. Additionally, we will explore multi-omics fusion techniques to fully leverage diverse datasets. Concurrently, we will assess imputation methods to further diminish the impact of data gaps in omics datasets.
